# Evaluating the Impact of Care Community Coaching Intervention: A Cluster Randomized Controlled Trial

**DOI:** 10.1093/geroni/igaf122.4242

**Published:** 2025-12-31

**Authors:** Boeun Kim, Basia Belza, Shawn Johnson, Emily Waddington, Walter Moczygemba, Lorna Prophater

**Affiliations:** University of Iowa, Iowa City, Iowa, United States; University of Washington, Seattle, Washington, United States; The Alzheimer’s Association, Chicago, Illinois, United States; Alzheimer’s Association, Chicago, Illinois, United States; University of North Carolina at Chapel Hill, Chapel Hill, North Carolina, United States; Alzheimer’s Association, Chicago, Illinois, United States

## Abstract

Within care communities, including nursing homes and assisted living settings, person-centered dementia care, highlighted by the 2018 Alzheimer’s Association Dementia Care Practice Recommendations (DCPR), is the foundation of quality care and improving staff outcomes. A cluster randomized controlled trial was conducted from November 2022 to April 2025 to evaluate a six-month Care Community Coaching intervention, delivered primarily in person, to enhance person-centered dementia care in alignment with the DCPR. A total of 77 care communities were randomized, with 434 staff members participating—227 from 38 intervention communities and 207 from 39 control communities. Outcomes included DCPR adoption, employee satisfaction, person-centered care practices, and dementia care confidence, measured post-intervention and at three-month follow-up. Generalized Estimating Equations were used to estimate intervention effects. Findings indicate that care coaching increased communities’ adoption of the DCPR by an average of 20.0%. Additionally, communities participating in care coaching showed statistically significant improvements in employee satisfaction, and staff perceptions of the delivery of individualized care and workplace practices. No statistically significant impacts on staff perceptions of caregiver-resident relationships or dementia care confidence were noted. Findings provide direction for future research and intervention development, including examining the impact of coaching on resident quality outcomes, and incorporating skills training into future models. Collectively, findings provide evidence of the effectiveness of a Care Community Coaching Program in DCPR adoption and improving staff outcomes and person-centered practices, offering a practical path towards improving the lived experience of residents and staff in care communities across the United States.

